# New Insights into Congenital Diaphragmatic Hernia – A Surgeon’s Introduction to CDH Animal Models

**DOI:** 10.3389/fped.2014.00036

**Published:** 2014-04-29

**Authors:** Priscilla Pui Lam Chiu

**Affiliations:** ^1^Division of Pediatric General and Thoracic Surgery, Department of Surgery, The Hospital for Sick Children, University of Toronto, Toronto, ON, Canada

**Keywords:** congenital diaphragmatic hernia, pulmonary hypoplasia, genetic models, teratogen, fetal lamb, fetal surgery, fetal tracheal occlusion

## Abstract

In recent decades, new research into the developmental defects and pathophysiological basis of congenital diaphragmatic hernia (CDH) has revealed opportunities for the development of innovative therapies. Importantly, the use of animal models to represent this anomaly in the laboratory has resulted in the discovery of many important genetic, epigenetic, and other molecular contributors to this condition. In this review, the most commonly used and newly devised animal models of CDH are presented to familiarize the reader with the latest innovations in the basic sciences.

## Introduction

Pediatric surgeons continue to face the challenge of treating a variety of congenital anomalies and neonatal disorders for which the etiology and pathophysiology are poorly understood and under-studied. In addition to the fact that many of these defects are rare in overall incidence and lack a known genetic cause, there has been a general dearth of biological or animal models to specifically study the conditions that result from developmental failures. However, for congenital diaphragmatic hernia (CDH), there has been much research effort and productivity. Over the past four decades, an increasing number of animal models – small and large animal, teratogenic, and experimental, have been employed to give scientists and clinicians the basis toward a better scientific understanding of this condition, we treat surgically. In this paper, I will provide a brief and updated summary of the currently available animal models for CDH, the history of their use, discoveries made using these models and highlight new insights for future studies.

## Congenital Diaphragmatic Hernia

Congenital diaphragmatic hernia is a rare birth defect affecting 1 in 2500 live births (1). Surgeons have been intimately involved in the treatment of this defect for over 60 years since Gross first reported his repair of a CDH neonate (2). While CDH overall mortality has decreased to 20% or less in the last decade (3, 4) compared to over 50% mortality of the previous decades owing largely to improvements in postnatal resuscitation and lung-preservation management (5, 6), mortality remains a significant outcome and long-term morbidity for survivors is common (3, 7, 8). Unequivocally, the greatest challenge for the past six decades in the management of CDH patients has been the inability to post-natally reverse the severity of lung hypoplasia affecting both lungs and not just on the side ipsilateral to the hernia (9). The cardiorespiratory morbidity for CDH survivors can persist well beyond the neonatal period. Fortunately, in the past three decades this anomaly has garnered great prominence and attention from both basic science and clinical research communities resulting in new knowledge about this condition and the promise of clinical interventions aimed at correcting the lethal lung defects.

To perform this research, scientists have utilized rodent models (teratogenic and genetic) and the experimental large animal (fetal lamb) model. These two models have been functionally although not exclusively complementary in their research applications. The teratogenic rodent model has a long history and is widely used to determine the fundamental developmental and physiological defects of CDH, specifically in dissecting the molecular and cellular features of CDH in order to understand their effects on diaphragm, lung, and vascular development (10). In parallel, the experimental fetal lamb model pioneered by de Lorimier et al. (11) in San Francisco and Haller et al. (12) in Baltimore has been adopted for studies of the CDH-affected lung and the surgical interventions to reverse these defects. Most importantly, its use has been instrumental for the advances in the field of fetal surgery including fetal tracheal occlusion for the prenatal intervention of high-risk CDH currently under clinical trial (13). Undoubtedly, this congenital anomaly has become the “poster child” for the bench-to-bedside crosstalk between basic and clinical researchers so that new discoveries can be translated into clinical interventions to improve CDH patient outcomes. With the pace of this research accelerating, the clinical benefits stemming from the research performed using these CDH animal models will continue to be realized in the near future.

## Teratogenic Model for CDH

The most commonly used teratogenic model for CDH is the nitrofen model. Nitrofen (also known as NIP in Japan and niclofen in Canada), is 2,4-dichloro-1-(4-nitrophenoxy)benzene, a Protox-inhibiting herbicide of the diphenyl ether class. Its use has been banned in Europe, Canada, and the United States since the mid-1990s for its documented carcinogenic effects on rodents (liver and pancreatic adenocarcinomas) and is considered a Group 2B class carcinogen (possibly carcinogenic to humans) (14, 15). It was identified as a teratogen in studies with rodent dams where pups were born cyanotic, exhibited respiratory distress, and died shortly after birth (16). The teratogenic effect of nitrofen is exerted mainly during the critical period of organogenesis (17). As a result, timed matings are required for the model and the herbicide is orally fed at a dose of 50 mg/kg/day or higher between E8–9 for mice and E8–12 for rats (correlating with human gestational age of 4–6 weeks), producing hypoplastic lungs and a spectrum of diaphragmatic defects in the pups similar to the human disorder (18, 19). Interestingly, the timing of teratogen exposure also affects CDH laterality, as earlier exposure increases the frequency of left-sided defects whereas administration later in gestation (E10–12) increases right-sided incidence (10). Not all of the pups are affected with CDH and, unlike the human disease, the litter will also have pups affected by other developmental abnormalities of the kidneys, heart, and skeleton (19, 20).

Initial studies using the nitrofen model were particularly fruitful in debunking embryological myths surrounding CDH pathogenesis, with the most detailed studies coming from John Greer’s group. While it had been speculated that CDH development resulted from the failure of the pleuroperitoneal canal to close in mid-gestation thereby causing the diaphragmatic sequelae, the nitrofen model revealed that CDH occurs early (17) from developmental abnormalities of the pleuropulmonary fold (PPF), specifically it is a muscular mesenchymal component (21, 22). Whether this finding applied to the human disease or specifically to this model of CDH pathogenesis was not proven. However, given that genetic models of CDH, such as the mouse models disrupting the expression of the Wilm’s Tumor 1 (WT1^−/−^) or the chicken ovalbumin upstream promoter-transcription factor II (COUP-TFII) gene (see the section below) showed similar changes in PPF morphology, there appears to be good evidence that similar changes underlie the human defect.

With access to the earliest stages of CDH pathogenesis in this teratogenic model, multiple developmental pathways have been implicated as the mechanism(s) driving nitrofen-induced CDH. Initial observations suggested that nitrofen was thyromimetic and exerted its teratogenic effects by disrupting the maternal–fetal thyroid hormone pathway through suppression of maternal TSH levels (23). However, subsequent studies showed direct delivery of the nitrofen compound rather than maternally produced active metabolites into the fetus through the maternal–fetal circulation, indicating the potential for direct effects of nitrofen on fetal diaphragm and lung development (24). More recent studies have also implicated the retinoic acid pathway as significantly altered by fetal nitrofen exposure (see the section below). Additionally, nitrofen may also exert epigenetic effects in lung development as miR-200b expression is decreased in nitrofen-induced lung hypoplasia (25). The extent of the molecular and developmental pathways implicated as mechanisms in nitrofen-induced CDH is beyond the scope of this review but is well summarized in a recent publication (26). Finally, the teratogenic effects of nitrofen do not appear to be limited to airway and diaphragm morphogenesis, as changes in pulmonary lymphatics (27), innervation (28–30), and the mesenchyme itself (31) were also observed in the nitrofen model. By interrogating the underlying molecular and genetic pathways occurring at the earliest stages of nitrofen-induced CDH pathogenesis, investigators are now devising therapies to minimize the severity of pulmonary hypoplasia and validating those therapies using this pre-clinical model (32–34).

For completeness, it should be noted that other chemicals have also been identified as teratogens causing CDH. One is 4-biphenyl carboxylic acid (BPCA), a metabolite of AH23848, a thromboxane-A_2_ receptor antagonist originally developed to inhibit platelet aggregation (35). Bisdiamine, or *N,N ′*-octamethylenebis (dichloroacetamide), is a spermatogenesis inhibitor whose teratogenic effects in addition to CDH include cardiovascular anomalies (36). SB-210661, a benzofuranyl urea derivative developed by GlaxoSmithKline, is a potent 5-lipoxygenase inhibitor developed as an anti-inflammatory agent for coronary artery disease. All three compounds were found to cause CDH in rats similar to nitrofen (37). Importantly, these agents, including nitrofen (38), inhibit retinal dehydrogenase-2 (RALDH-2), a key enzyme for the production of retinoic acid, suggesting that disruption of the retinoid signaling pathway (including vitamin A deficiency) contributes to CDH pathogenesis. Conversely, augmentation of vitamin A levels may also reverse this defect (39), leading to the tantalizing possibility that the earliest interventions to prevent CDH-related defects may be as simple as vitamin supplementation. To further dissect the role of the retinoid acid pathway in CDH pathogenesis, genetic models were the next logical tools for these investigations.

## Genetic Models of CDH

Whether by intention or serendipity, genetic models of CDH became available as molecular techniques for gene silencing or over-expression grew into wider application. While the majority of human CDH cases are not associated with known genetic defects [I will refer the reader to reference (40) for an excellent review of the genetic factors in human CDH], multiple genetic models have been associated with CDH as part of their phenotype. Although the specific effect of retinoid pathway inhibition on CDH development was not revealed until recently, the disruption of both retinoic acid receptor α and β genes, thus disrupting the entire retinoic acid signaling pathway, resulted in CDH (41) and confirmed previous reports implicating the role of vitamin A deficiency in CDH development (42, 43). Not surprisingly, disruption of other genes involved in the retinoic acid pathway has also resulted in CDH amongst other defects in organogenesis (44, 45).

Genes encoding hormone receptors overlapping with the retinoid pathway have also yielded the CDH phenotype when their expression has been disrupted. The COUP-TFII gene, also known as NR2F2, is a transcriptional protein belonging to the steroid/thyroid hormone receptor superfamily whose expression is regulated by retinoids and, in turn, include among its functions the regulation of gene transcription by modulation of retinoic acid receptor heterodimerization (46, 47). Of note, the COUP-TFII gene is on chromosome 15q26 – a region recurrently deleted in CDH patients (48, 49), thus making it the candidate gene. Mice with tissue-specific ablation of this gene also exhibit CDH defects (50).

The role of genes less obviously associated with human CDH has also been revealed through genetic models. Mutations of the Wilm’s Tumor 1 gene, *WT1*, in two cases of human CDH-associated with Denys–Drash syndrome have been reported (40). Abrogated expression of *WT1* in mice was found to be associated with fetal CDH development (51) with its effects appearing to be exerted through the common retinoid pathway (52). In some cases, diaphragmatic defects resulting from genetic defects bear less resemblance to the classic human condition. Genes that encode the SLIT family of proteins involved in a diverse array of neuronal cells also appear to affect diaphragm development although slightly different from the classic Bochdalek hernias when their expression is disrupted. Mice harboring the disrupted *slit3* gene develop a central CDH similar to Morgagne hernias rather than Bochdalek hernias (53). Friend of GATA 2 (FOG2) encodes a zinc finger protein (54) that primarily interacts with GATA4, a member of a family of DNA-binding proteins so named because they recognize the GATA motif in the promoter regions of many genes (55). FOG2–GATA4 interaction modulates gene expression during many developmental processes including heart morphogenesis. Mice harboring a *fog2* mutation were found to have diaphragmatic defects in the posterior and peripheral aspects (56) while mice carrying a single copy of the mutated *gata4* developed heart, lung, and diaphragm defects (57). Interestingly, the chromosomal regions where the human FOG2 and GATA4 genes are located have been found to be deleted in some CDH patients (40).

## Experimental Model for CDH

While the CDH rodent models are scaled and optimized to uncover early developmental and genetic derangements contributing to CDH, large animal models for CDH are scaled for life-sized interventions of the affected lung or diaphragm for direct translation to the clinical domain. As large animal models of CDH require diaphragmatic defects to be created during early stages of fetal development, this model cannot be used to study the earliest origins of the CDH diaphragmatic defect that is better served by the rodent models. The fetal lamb is the most commonly used experimental model but some researchers have also used rabbits for similar studies (58).

One of the pivotal reasons for choosing the fetal lamb model for CDH was that there was already a large body of literature on fetal lamb pulmonary physiology prior to its adoption as a CDH model. With this background, the fetal lamb model for CDH has aided the study of ventilator-related lung damage observed in CDH and the development of fetal interventions aimed at arresting or reversing CDH-associated pulmonary hypoplasia. As the fetal lamb model was more expensive and time-consuming for CDH research compared to rodent models, most investigations focused exclusively on defining the pulmonary effects of CDH or fetal ventilation strategies (59–61). To create this model (and technical details varied between laboratories), the fetal lamb is delivered out of the ewe between 80 and up to 110 days’ gestation with the gravid ewe under full general anesthesia. The technique requires positioning the hysterotomy directly over the fetal thorax by palpation of the uterus in order to minimize the size of the uterine incision. The chest of the fetal lamb is delivered out of the wound for the incision to be made in the 11th intercostal space, thus exposing the fetal diaphragm. The diaphragm is incised and abdominal viscera are introduced into the thorax. The thorax is closed and the hysterotomy repaired. Planned delivery of the treated fetal lambs is required as ventilatory support immediately post-delivery is necessary. In initial studies where fetal interventions required repeat open fetal surgery, the overall survival of the fetal lambs was disappointingly low as the primary procedure to create the CDH was performed earlier in gestation and repeat hysterotomy created challenges including pre-term labor and stillbirths (62, 63). However, the hard lessons learned from these studies brought about more ingenious ways to circumvent these obstacles, including the application of tocolytic protocols and the eventual use of minimal access approaches for fetal repair.

Twenty-five years after he first started his laboratory studies in the fetal lamb model for CDH, the first randomized controlled trial of fetal tracheal occlusion for the treatment of high-risk CDH was reported by Harrison et al. in 2003 (64). While the outcome of fetal tracheal occlusion for high-risk CDH infants did not appear significantly different from the conventionally treated cohort, the trial represented the culmination of the many years of laboratory-based research, scientific interactions, and optimization of techniques using the experimental fetal lamb model in pre-clinical studies (60, 65–67). Consequently, clinicians treating other congenital anomalies with open fetal surgery now have the innovative techniques and clinical tools to support their procedures (68). The fetal lamb model is now widely used as a pipeline for the refinement of fetoscopic and open fetal surgical techniques for trainees (69).

## Conclusion

Animal models for CDH have generated vast troves of data in diverse areas of science and medicine. Rodent and fetal lamb models for CDH have encouraged crosstalk between geneticists, developmental biologists, and clinicians to discover new knowledge and create new treatment targets for fetal interventions, particularly in arresting or reversing the devastating pulmonary sequelae of CDH. The field of fetal surgery owes a debt of thanks to CDH disease and the research teams who first imagined fetal surgery as a treatment option. The past use of these animal models has yielded exciting results, spawning clinical trials and novel therapies for CDH. There is hope and excitement for other congenital anomalies that similar use of animal models will also bring such fruitful results for our patients.

## Conflict of Interest Statement

The author declares that the research was conducted in the absence of any commercial or financial relationships that could be construed as a potential conflict of interest.
